# Is There a Seasonal Difference in the Detection of Trichomonas vaginalis by Cervical Cytology?

**DOI:** 10.1100/tsw.2003.10

**Published:** 2003-03-17

**Authors:** Sydney Shrader, Enrique Hernandez, John Gaughan

**Affiliations:** ^1^ Medical Corps, U.S. Navy, Temple University School of Medicine, 3420 N. Broad St., Philadelphia, PA 19140, USA; ^2^ Department of Obstetrics, Gynecology and Reproductive Sciences, Temple University School of Medicine, 3420 N. Broad St., Philadelphia, PA 19140, USA; ^3^ Department of Biostatistics, Temple University School of Medicine, 3420 N. Broad St., Philadelphia, PA 19140, USA

**Keywords:** Trichomonas, cytology, cervix, vagina, Papanicolaou, prevalence

## Abstract

This objective of our study was to determine the prevalence of a Trichomonas vaginalis diagnosis in routine Papanicolaou smears and whether it is seasonal. We reviewed the diagnosis rendered for 93,681 Papanicolaou smears evaluated at a medical school hospital laboratory between 1992 and 1997. The occurrence of a diagnosis of T. vaginalis was analyzed by year, by quarter, and by month using a generalized linear regression model. The prevalence of a T. vaginalis diagnosis during the 6-year study period was 3.1%. The between-month and between-quarter comparisons of prevalence were not statistically different. In the population reported here, the prevalence of a Papanicolaou smear diagnosis of T. vaginalis was low and no seasonal difference in making this diagnosis was identified.

## INTRODUCTION

*Trichomonas vaginalis* infects approximately 180 million people worldwide annually[1]. Its prevalence varies among populations. A study of patients attending family planning clinics showed that 5% harbored the parasite[2]. Higher prevalence exists among pregnant women (20–25%), symptomatic women (25–50%), and sexually transmitted disease (STD) clinic patients (20–30%)[3]. Risk factors for *T. vaginalis* include multiple sexual partners, low socioeconomic status, African American race, concurrent gonorrhea, any previous STD or nonuse of barrier contraception[4]. Women using hormonal contraceptives experience a lower rate of trichomoniasis when compared to women who do not use a birth control method. Other factors, such as age and chlamydial infection are not as strongly linked to *T. vaginalis*.

While collecting Papanicolaou smears for a study of cytologic sampling techniques, Eisenberger et al.[5] found a higher percentage of slides with trichomonads in spring and winter months (9.8%) compared to autumn and summer (5.8%). The overall rate of *T. vaginalis* detection in Papanicolaou smears in this study population was 7.8%. Although the seasonal difference may have arisen from the study design, it raised the possibility that season may play a role in trichomonal infection, as it has been suggested to do in gonorrhea[6]. The present study does not intend to analyze the accuracy of the diagnosis of *T. vaginalis* by cervical cytology. The objective was to determine the prevalence of this diagnosis in routine Papanicolaou smears and whether, as suggested by the Eisenberger et al. study[5], the season of the year affects the proportion of Papanicolaou smears diagnosed as containing *T. vaginalis*. If a significant seasonal variation is confirmed, it would warrant further investigations as to its cause and significance.

## MATERIALS AND METHODS

We retrospectively reviewed the results of 93,681 Papanicolaou smears evaluated by the Department of Pathology and Laboratory Medicine at the Medical College of Pennsylvania Hospital from January 1, 1992 to December 31, 1997. We only reviewed a list of cytologic diagnoses without patient-identifying information and the study was exempt from review by the Institutional Review Board. Demographic information was not available for review. Although one half of the Papanicolaou smears were obtained from women attending the hospital clinics, the other half came from patients seen in the private practice offices of practitioners through out the Philadelphia metropolitan area. Almost all patients seen in the hospital's obstetrics and gynecology clinics were low-income African American women while those seen in the private practice setting were middle-class Caucasian women. The following approach for obtaining cervical cytology was routinely used by practitioners in the Philadelphia area during the study period. An Ayre (wood or plastic) spatula and an endocervical brush were utilized. In pregnant women a saline-moistened cotton-tipped applicator was used instead of the endocervical brush. The sample was smeared on a single glass slide and immediately sprayed with fixative.

In-house certified cytotechnologists reviewed all Papanicolaou smears included in this study. A cytopathology fellow and a faculty cytopathologist examined abnormal slides. For quality control, a cytotechnology supervisor reviewed 10% of slides with a diagnosis of normal. The laboratory staff did not change during the 6-year study period. It consisted of the same three cytotechnologists and the same four cytopathologists.

In this laboratory the cytologic diagnosis of *T. vaginalis* was based on the presence of intact organisms using strict criteria. Well-preserved trichomonads had to be seen. On Papanicolaou smears these appear oval- to pear-shaped, stain blue, and contain a small nucleus (Fig. 1). The cell may also contain reddish cytoplasmic granules[7].

The occurrence of *T. vaginalis* was analyzed in Papanicolaou smears by year, by quarter [winter (Jan.-March); spring (April-June); summer (July-Sept.); and autumn (Oct.-Dec.)], and by month using a generalized linear regression model (Poisson regression). This method provides for an individual patient having multiple Papanicolaou smears during the observation period (nonindependent events). Due to the redundancy (i.e., collinearity) in the data when classified by month, quarter, and year, each was analyzed separately.

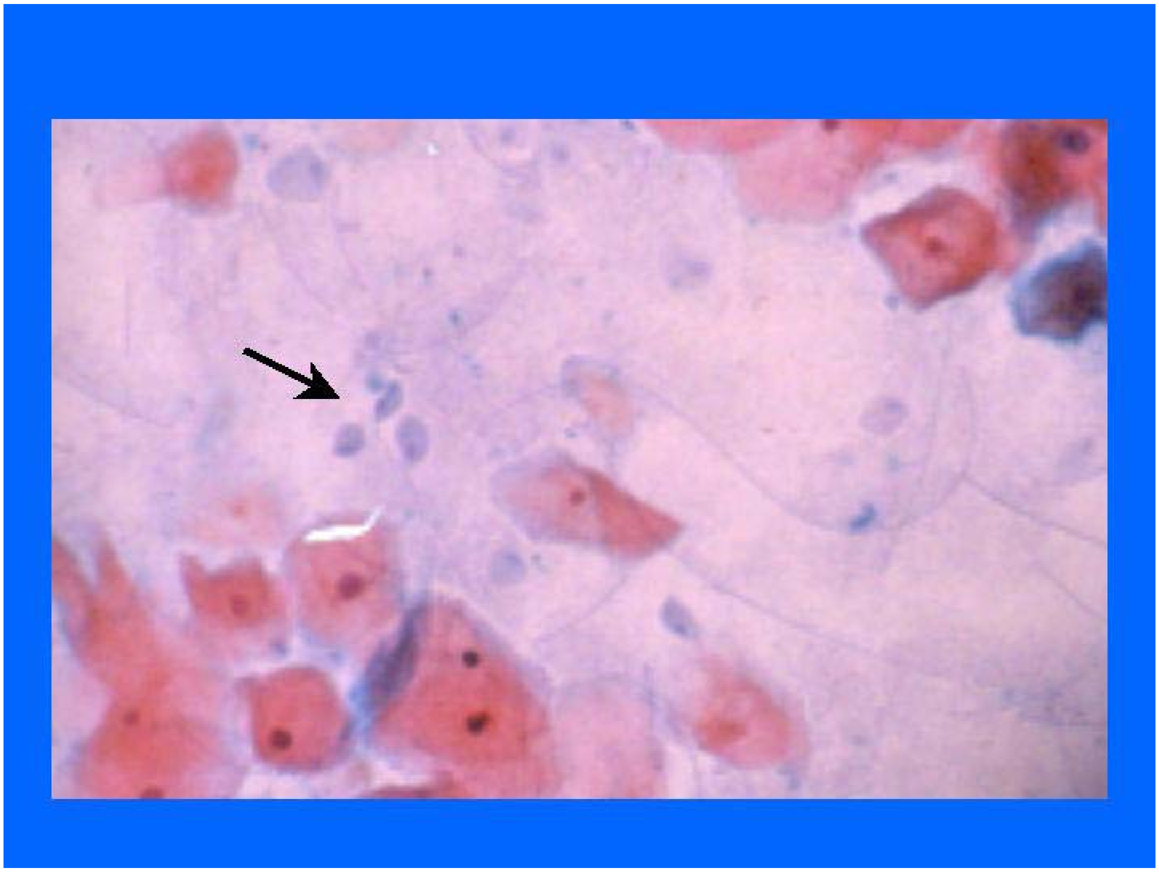

**FIGURE 1**. Papanicolaou smear showing many superficial and intermediate cervical squamous cells and several trichomonads. Three *T. vaginalis* organisms are seen almost in the center (at arrow) of the figure. They are oval- to pear-shaped, stain blue, and contain a small nucleus.

## RESULTS

For the 6-year study period, 2,927 (3.1%) of the 93,681 Papanicolaou smears evaluated were diagnosed as containing *T. vaginalis* (Table 1). There was not a statistically significant difference in the between-month ($p = 0.55$) and between-quarter ($p = 0.14$) comparisons. The between-year comparisons showed that the diagnosis of *T. vaginalis* was made less frequently in 1996 (2.3%) than in other years ($p < 0.01$). For the other years there were no statistically significant differences in the frequency of the diagnosis.

**TABLE 1**
**Total Number of Papanicolaou Smears with *T. vaginalis* vs. Total Number of Smears by Year, Month, and Quarter (qtr)**

| YEAR | MONTH | QTR | TRICH | TOTAL | YEAR | MONTH | QTR | TRICH | TOTAL |
|---|---|---|---|---|---|---|---|---|---|
| 1992 | JAN | 1 | 48 | 1353 | 1995 | JAN | 1 | 46 | 1044 |
| 1992 | FEB | 1 | 46 | 1135 | 1995 | FEB | 1 | 43 | 840 |
| 1992 | MAR | 1 | 59 | 1279 | 1995 | MAR | 1 | 25 | 1023 |
| 1992 | APR | 2 | 48 | 1257 | 1995 | APR | 2 | 33 | 922 |
| 1992 | MAY | 2 | 48 | 1223 | 1995 | MAY | 2 | 35 | 1007 |
| 1992 | JUN | 2 | 55 | 1424 | 1995 | JUN | 2 | 32 | 972 |
| 1992 | JUL | 3 | 49 | 1588 | 1995 | JUL | 3 | 34 | 964 |
| 1992 | AUG | 3 | 35 | 1304 | 1995 | AUG | 3 | 50 | 1315 |
| 1992 | SEP | 3 | 58 | 1431 | 1995 | SEP | 3 | 46 | 1444 |
| 1992 | OCT | 4 | 53 | 1469 | 1995 | OCT | 4 | 39 | 1941 |
| 1992 | NOV | 4 | 55 | 1412 | 1995 | NOV | 4 | 35 | 1895 |
| 1992 | DEC | 4 | 43 | 1445 | 1995 | DEC | 4 | 30 | 1484 |
| 1993 | JAN | 1 | 60 | 1415 | 1996 | JAN | 1 | 46 | 1479 |
| 1993 | FEB | 1 | 38 | 1151 | 1996 | FEB | 1 | 38 | 1941 |
| 1993 | MAR | 1 | 48 | 1339 | 1996 | MAR | 1 | 49 | 2507 |
| 1993 | APR | 2 | 52 | 1398 | 1996 | APR | 2 | 59 | 3043 |
| 1993 | MAY | 2 | 42 | 1369 | 1996 | MAY | 2 | 44 | 3165 |
| 1993 | JUN | 2 | 51 | 1257 | 1996 | JUN | 2 | 32 | 2839 |
| 1993 | JUL | 3 | 47 | 1094 | 1996 | JUL | 3 | 51 | 2782 |
| 1993 | AUG | 3 | 49 | 1155 | 1996 | AUG | 3 | 58 | 1710 |
| 1993 | SEP | 3 | 46 | 1039 | 1996 | SEP | 3 | 43 | 1027 |
| 1993 | OCT | 4 | 44 | 1328 | 1996 | OCT | 4 | 44 | 1220 |
| 1993 | NOV | 4 | 31 | 1288 | 1996 | NOV | 4 | 33 | 991 |
| 1993 | DEC | 4 | 35 | 1357 | 1996 | DEC | 4 | 22 | 816 |
| 1994 | JAN | 1 | 34 | 870 | 1997 | JAN | 1 | 29 | 912 |
| 1994 | FEB | 1 | 38 | 1004 | 1997 | FEB | 1 | 39 | 838 |
| 1994 | MAR | 1 | 32 | 1229 | 1997 | MAR | 1 | 26 | 768 |
| 1994 | APR | 2 | 42 | 1336 | 1997 | APR | 2 | 22 | 774 |
| 1994 | MAY | 2 | 41 | 1252 | 1997 | MAY | 2 | 30 | 876 |
| 1994 | JUN | 2 | 55 | 1274 | 1997 | JUN | 2 | 25 | 796 |
| 1994 | JUL | 3 | 42 | 1184 | 1997 | JUL | 3 | 28 | 831 |
| 1994 | AUG | 3 | 42 | 1348 | 1997 | AUG | 3 | 33 | 771 |
| 1994 | SEP | 3 | 39 | 1158 | 1997 | SEP | 3 | 23 | 697 |
| 1994 | OCT | 4 | 30 | 1266 | 1997 | OCT | 4 | 36 | 815 |
| 1994 | NOV | 4 | 39 | 1151 | 1997 | NOV | 4 | 18 | 614 |
| 1994 | DEC | 4 | 39 | 1104 | 1997 | DEC | 4 | 22 | 632 |

## DISCUSSION

*T. vaginalis*, a protozoan, is the most common parasite infecting the vagina[8]. In addition to vaginitis, *T. vaginalis* may cause exocervicitis, or urethritis (in both men and women). However, 9–56% of women (and many men) harboring *T. vaginalis* are asymptomatic. This infection is almost always contracted sexually, although nonvenereal transmission may occur extremely rarely. Several serotypes exist which differ in features such as surface molecules, size, and, in experimental settings, virulence[9,10]. Higher rates of trichomoniasis are associated with multiple sexual partners, previous STD infection (especially gonorrhea), and low socioeconomic status. Of women whose male partners acquire *T. vaginalis*, 66–100% also carry the organism[10].

Papanicolaou smears have been demonstrated to be only 60–70% sensitive for detecting trichomonads[8,9]. In addition, the relatively high false-positive rate for *T. vaginalis* on cytologic smears has been a drawback in basing diagnosis and treatment on this test[8,11]. Over 20% of gynecologic and 30% obstetric patients would undergo unnecessary treatment when a positive Papanicolaou smear result is not confirmed by another test[11]. Therefore, when using Papanicolaou smears to monitor the rate of *T. vaginalis* infection, the potential for false positive readings, even by experienced cytopathologists, should be taken into consideration. Degenerated cells and bare nuclei in inflammatory and atrophic smears can be confused with trichomonads. Even with this shortcoming, culture for *T. vaginalis* is expensive and not readily available while the Papanicolaou smear is routinely performed. In addition, a repeat smear following and initial positive result has a sensitivity and specificity for *T. vaginalis* of 86 and 83%, respectively[12]. The objective of this study was not to determine the accuracy of cervical cytology for diagnosing *T. vaginalis* infection, but to determine the frequency of this diagnosis in routine Papanicolaou smears.

The overall prevalence of *T. vaginalis* in Papanicolaou smears in our study is lower than that seen in some studies, but similar to that reported from family planning clinics[2]. Our investigation included slides sent from physicians' offices throughout the Philadelphia metropolitan area in addition to those from the medical school hospital clinics. This population included groups with fewer risk factors for *T. vaginalis*, which may account for the relatively low prevalence we observed.

Among a population of mainly low-income African American women visiting the family planning, gynecology, and colposcopy clinics of an inner-city university hospital, Eisenberger et al.[5] found that the overall prevalence of *T. vaginalis* in Papanicolaou smears was 7.8% and higher in winter and spring. However, the study sample was much smaller than that reported here. In addition, the method by which the cytology sample was obtained was different in different months and the samples were obtained over an interval of less than 1 year. We questioned whether the seasonality of *T. vaginalis* diagnosis in cervical cytology specimens was real or due to the study design. If seasonality in *T. vaginalis* diagnosis was identified, it could warrant further investigations as to its cause and significance.

Our investigation, over a longer period of time, did not reveal seasonality to the diagnosis of *T. vaginalis* by Papanicolaou smears. In this analysis of almost 100,000 cervical-vaginal cytology specimens examined by one laboratory over a period of 6 years we found that the diagnosis of *T. vaginalis* was made in 3% of the specimens. The only statistically significant difference found was that in 1996 the diagnosis of *T. vaginalis* was made in 2.3% of the smears. This small difference, although statistically significant, is not clinically significant.
